# An Isolated Manubrium Fracture in a Young Football Player: A Case Report

**DOI:** 10.7759/cureus.81336

**Published:** 2025-03-28

**Authors:** Hassan Abou Adma, Huy Tran, Martin Holguin, Daniel Romanelli

**Affiliations:** 1 Medicine and Surgery, Saint James School of Medicine, McAllen, USA; 2 Orthopedic Surgery, South Texas Health System, Edinburg, USA; 3 Orthopedic Surgery, Doctors Hospital at Renaissance, Edinburg, USA

**Keywords:** conservative management of fractures, contact sports injury, eccentric training recovery, football injury, high-impact sports injury, isolated manubrium fracture, non-displaced manubrium fracture, sternal fractures, trauma-induced manubrium fractures

## Abstract

This case report highlights a rare occurrence of an isolated manubrium fracture in a 17-year-old high school football quarterback. Such fractures are uncommon in athletes despite the high-impact nature of contact sports. The injury was sustained during a tackle and confirmed via imaging as an oblique fracture to the right lateral manubrium with minimal posterior displacement. No reduction or surgical intervention was required, and the patient was managed conservatively with rest, analgesia, and physical therapy, including eccentric training to aid recovery and strength rebuilding.

The report discusses mechanisms and the risk of manubrium fractures in contact sports, emphasizing the role of direct trauma and repetitive stresses as etiologies. Imaging modalities such as X-ray and CT scans are essential for accurate diagnosis and ruling out associated injuries to vital thoracic structures. Management ranges from non-operative approaches for non-displaced fractures to surgical interventions for severe cases with rehabilitation focusing on pain management, functional recovery, and return-to-play readiness.

Recognizing and managing rare sternal injuries in athletes is important, with an emphasis on a multidisciplinary approach to ensure optimal recovery and safe reintegration into sports. Tailored rehabilitation with incorporation of eccentric training is effective in enhancing recovery while mitigating the risk of re-injury.

## Introduction

Sternal fractures are rare injuries accounting for less than 0.5% of all fractures [1]. These injuries are most commonly associated with direct traumatic injuries, particularly in high-impact scenarios such as motor vehicle accidents, with a reported sternal fracture incidence of 3% to 8%. Manubrium fractures, a specific type of sternal fracture, are extremely rare, accounting for approximately 15% of all sternal fractures due to traumatic injuries [2]. Sports-related injuries, both in contact and non-contact sports, can also lead to manubrium fractures. While specific cases related to contact sports causing manubrium fractures are less documented, the nature of these sports, with their physical collisions and potential for high-impact trauma, logically extends to injuries to the manubrium. An interesting case of a manubrium sterni fracture was reported in a young man as a complication of bodybuilding exercises, a non-contact sport [3]. Repetitive stress and torque exerted on the manubrium during specific activities can also lead to fractures [4]. Multiple fractures, including manubrium fractures, can occur secondary to the tonic-clonic muscular contractions of a seizure [5].

## Case presentation

A 17-year-old male presented to our facility with complaints of sternum pain following an injury sustained during a football game. The patient, a senior in high school, plays the quarterback position. He had no significant previous medical history, prior surgical procedures, or history of traumatic injuries. The patient's injury occurred while playing football, specifically during a tackle on the field, and he subsequently landed hard on his left shoulder. Immediately upon impact, he experienced a sudden and sharp pain localized to the sternum area. Recognizing the severity of the injury, he was promptly brought to our facility for evaluation. At the time of presentation, the patient was found wearing an arm sling with a self-reported pain level of 4/10, which increases with movement of the right arm in abduction, adduction, flexion, extension, and external and internal rotation. Physical examination revealed no obvious swelling or deformities. There was tenderness over the medial clavicle at the region of the sternoclavicular joint upon palpation. The patient denied shortness of breath or swallowing problems. Neurovascular appeared intact distally. Vital signs were within normal limits.

**Figure 1:**
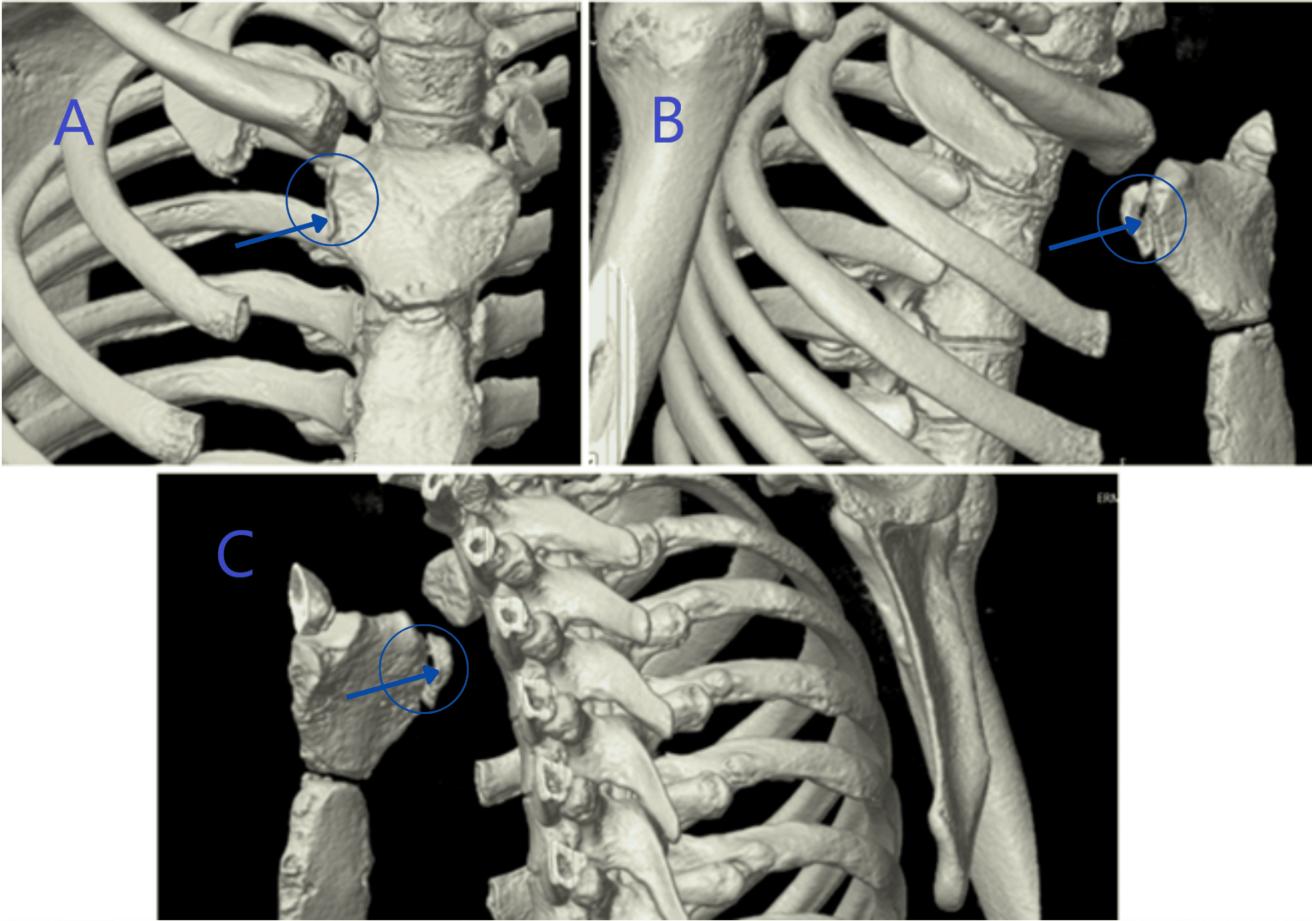
3D CT scans of the fractured manubrium A: coronal view highlighting the manubrium fracture (blue arrow); B: sagittal view highlighting the minimal displacement of the fracture (blue arrow); C: additional parasagittal view highlighting the minimal displacement of the fracture (blue arrow)

Imaging was ordered given the nature of the injury and the potential for associated complications. Computed tomography (CT) results, Figure 1, confirmed an acute fracture of the right lateral portion of the manubrium. No acute displacement fracture or dislocation of the right shoulder was noted. No reduction was needed due to the nature of the fracture, and the patient was advised to avoid contact sports for three to four months with regular periods of follow-up to track his healing progress.

## Discussion

**Figure 2:**
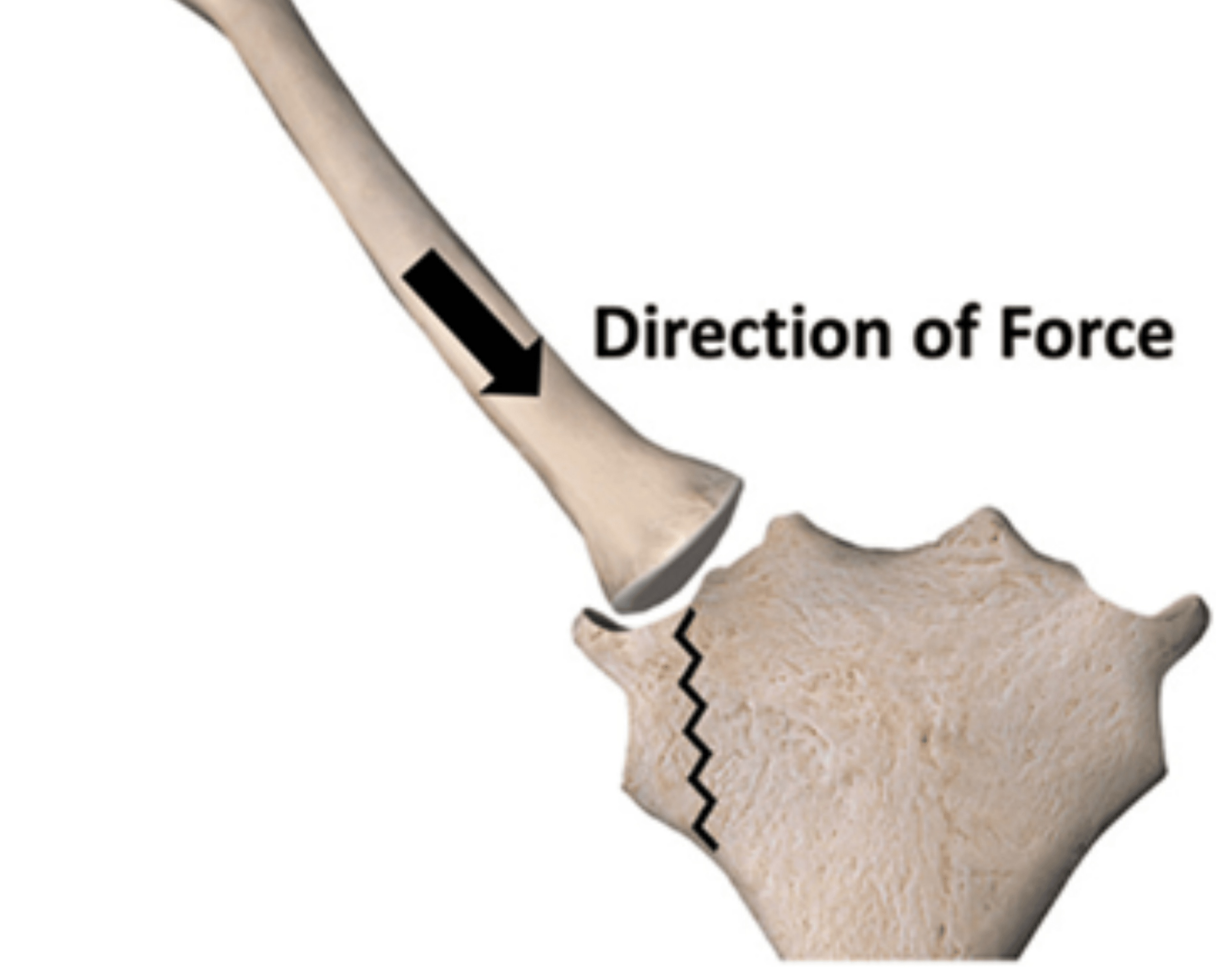
Proposed mechanism of injury Reproduced from [7]; used under CC BY-NC-ND 4.0

In this report, we highlight a rare presentation of an isolated manubrium fracture in a football athlete. The patient's CT, Figure 1, confirmed the diagnosis of an oblique fracture with minimal posterior displacement of the right superolateral portion of the manubrium. We believe the mechanism of injury in this patient to be a posteromedial subluxation of the proximal clavicle leading to shearing of the superolateral portion of the manubrium with posterior deviation, Figure 2. Although rare, these fractures present a notable concern in young athletes engaged in contact sports. The sternum’s anatomical position and its role in protecting vital thoracic organs make such injuries particularly significant. The incidence of isolated manubrium fractures in athletes is rare when compared with other common occurrences, with certain fracture types being more prevalent. In collegiate athletes, the overall reported general fracture rate of 1.80 per 1000 athletes exposed during competitions than practice and 1.82 per 1000 in high school athletes, with 4.37 per 10,000 exposures due to football [6].

Due to the high-impact nature of contact sports, although rare, football and rugby players are susceptible to manubrium fractures resulting from direct trauma to the chest. A retrospective analysis conducted on 890 individuals demonstrated 154 patients with sternal fractures, of which 23 (14.9%) suffered fractures involving the manubrium, ranging from transverse fracture (47.9%), oblique fracture (39.1%), and combined fractures (13%) [2]. The majority of the fractures follow motor vehicle collisions (MVCs) through steering wheel impact, falling from heights greater than three meters, and hyperflexion/hyperextension trauma to the torso [8-10]. Notable cases involve a 20-year-old football player suffering a moderate chest collision leading to a sternal fracture and another case of a young bodybuilder developing a manubrium sterni stress fracture due to repetitive strain. Although it is noteworthy to highlight the vulnerability of sternal fractures from a direct impact, repetitive stresses and overuse in sports requiring heavy lifting and sustained exertion can also lead to such injuries [8-11].

The increased susceptibility of transverse as well as oblique fractures of the manubrium in football and rugby players is a result of direct trauma through the high-impact tackles the players face on a repetitive basis. Through rapid deceleration, the manubrium, with or without the involvement of the sternum, can undergo shearing in a transverse or oblique fashion depending on the nature of the forces experienced. Indirect trauma through forces from the clavicles, chin, and upper two ribs can lead to posterior dislocation of the manubrium. The manubrium has multiple articulations with the clavicles, first and second ribs, and the sternum; it is important to conduct a thorough examination to rule out associated injuries to posterior structures through the use of X-ray, CT, and magnetic resonance imaging (MRI), including the superior vena cava (SVC) and jugular veins, carotid arteries, trachea, esophagus, and the lung apices [2,7]. The most common associated injuries with manubrium and sternal fractures are concomitant rib fractures, pulmonary contusions, pneumothorax, and myocardial contusions [3,12].

The optimal management strategy for isolated manubrium fractures in athletes involves both operative and non-operative approaches depending on the severity and displacement of the fracture. For non-displaced fractures, conservative management with rest, analgesia, and physical therapy is typically recommended with a main focus on pain management and gradual return to normal activities [7,11]. In cases of displaced fractures, surgical intervention may be a necessity, and the utilization of intramedullary fixation has been shown to be successful in clinical outcomes and rapid resumption of activities in athletes with midclavicular fractures, suggesting a potential benefit for manubrium fractures as well [12,13]. By utilizing techniques similar to the Titanium Elastic Nail (TEN) system in athletes, it can result in successful clinical outcomes and a rapid resumption of sporting activities [13]. Adequate healing may take four to six weeks up to 12 weeks post-injury with additional rehabilitation enhancing the patient’s recovery [12].

The return-to-play criteria look at functional recovery, rehabilitation progress, pain assessment, and previous injury history [14]. Prior to engagement in sporting activities, the athlete should have a full, painless range of motion and restored muscle strength with the completion of a progressively comprehensive functional exercise program specifically including eccentric training [15,16,17]. Pain levels at the time of injury and areas of tenderness from the initial evaluation should be assessed to estimate the time to return to play. A history of previous injury should be taken into consideration in the athlete’s progression to sport, as previous injury is a risk factor for future re-injury [18,19].

Eccentric training plays a significant role in rehabilitation for isolated manubrium fractures as it is used for enhancing muscle strength, improving physical performance, and aiding in the recovery process [16,19]. It has been shown that eccentric training promotes increases in muscle mass and strength when compared to concentric training, particularly when performed at high intensities. Effectively performing eccentric exercises aids in strengthening the muscles of the chest, supporting the healing process, in addition to improving flexibility and pain reduction. Utilizing gradual load increases and controlled movements promotes adequate healing and minimizes injury by starting with low-intensity exercises and gradually increasing the load, which safely strengthens the chest muscles without overloading the fracture site. Studies have shown significant strength gains with improvements of 20% to 50% over a period of six to 12 weeks and a reduction in pain levels by 30% to 50% after consistent eccentric training [12,15,18].

## Conclusions

This report highlights the rare occurrence of an isolated manubrium fracture in a high school football athlete, emphasizing the importance of recognizing such injuries in contact sports. Sternal fractures, especially those involving the manubrium, are uncommon but can have significant implications for athletes. The mechanism of injury, in this case, was attributed to a posteromedial subluxation of the proximal clavicle, leading to shearing of the superolateral portion of the manubrium with posterior deviation. While the incidence of manubrium fractures is relatively low in athletes, especially football and rugby players, it is essential to be aware of the risks associated with high-impact tackles and collisions. This report also highlights the variety of fracture patterns observed in manubrium injuries, including transverse, oblique, and combined fractures, which can result from different mechanisms of trauma. It is important that diagnosis is confirmed through imaging techniques like X-ray, CT, and MRI, helping identify any associated injury to critical structures.

Management strategies for manubrium fractures vary based on the severity and displacement of the fractures. While intramedullary fixation shows promising outcomes, concomitant utilization of analgesia and physical therapy with a focus on eccentric training aids in pain management and gradual return to normal activities. Overall, a multidisciplinary approach involving careful diagnosis, appropriate management, and tailored rehabilitation is essential for optimal recovery and safe return to sports for athletes with manubrium fractures.

## References

[1] Klei DS, de Jong MB, Öner FC, Leenen LP, van Wessem KJ (2019). Current treatment and outcomes of traumatic sternal fractures-a systematic review. Int Orthop.

[2] Schulz-Drost S, Krinner S, Oppel P, Grupp S, Schulz-Drost M, Hennig FF, Langenbach A (2018). Fractures of the manubrium sterni: treatment options and a possible classification of different types of fractures. J Thorac Dis.

[3] Robertsen K, Kristensen O, Vejen L (1996). Manubrium sterni stress fracture: an unusual complication of non-contact sport. Br J Sports Med.

[4] Baker JC, Demertzis JL (2016). Manubrial stress fractures diagnosed on MRI: report of two cases and review of the literature. Skeletal Radiol.

[5] Gill JR, Murphy CG, Quansah B, Carrothers AD (2015). Seizure induced polytrauma; not just posterior dislocation of the shoulder. BMJ Case Rep.

[6] Swenson DM, Henke NM, Collins CL, Fields SK, Comstock RD (2012). Epidemiology of United States high school sports-related fractures, 2008-09 to 2010-11. Am J Sports Med.

[7] Bardos A, Sabhrawal S, Tytherleigh-Strong G (2021). Management of vertical sternal fracture nonunion in elite-level athletes. Orthop J Sports Med.

[8] Demir Benli M (2023). Stress fracture of the manubrium sterni during parallel bar dips. Phys Sportsmed.

[9] Douglas RJ (2008). Sternal fracture in an Australian Rules footballer. Med J Aust.

[10] FO AW (1957). Flexion-compression injury of the sternum. J Bone Joint Surg Br.

[11] Smith M, Lenehan B, O'Keefe D, Martin A (2001). Manubriosternal joint dislocation in contact sport. Emerg Med J.

[12] Cairns MA, Hasty EK, Herzog MM, Ostrum RF, Kerr ZY (2018). Incidence, severity, and time loss associated with collegiate football fractures, 2004-2005 to 2013-2014. Am J Sports Med.

[13] Jubel A, Andemahr J, Bergmann H, Prokop A, Rehm KE (2003). Elastic stable intramedullary nailing of midclavicular fractures in athletes. Br J Sports Med.

[14] Bonsignore-Opp L, Galivanche A, El Naga AN, Gendelberg D (2024). Return to play criteria after adult lumbar spinal fractures: a review of current literature and expert recommendations. Curr Rev Musculoskelet Med.

[15] Frizziero A, Trainito S, Oliva F, Nicoli Aldini N, Masiero S, Maffulli N (2014). The role of eccentric exercise in sport injuries rehabilitation. Br Med Bull.

[16] Bright TE, Handford MJ, Mundy P, Lake J, Theis N, Hughes JD (2023). Building for the future: a systematic review of the effects of eccentric resistance training on measures of physical performance in youth athletes. Sports Med.

[17] Hoenig T, Eissele J, Strahl A (2023). Return to sport following low-risk and high-risk bone stress injuries: a systematic review and meta-analysis. Br J Sports Med.

[18] Malliaropoulos N, Mendiguchia J, Pehlivanidis H, Papadopoulou S, Valle X, Malliaras P, Maffulli N (2012). Hamstring exercises for track and field athletes: injury and exercise biomechanics, and possible implications for exercise selection and primary prevention. Br J Sports Med.

[19] Roig M, O'Brien K, Kirk G, Murray R, McKinnon P, Shadgan B, Reid WD (2009). The effects of eccentric versus concentric resistance training on muscle strength and mass in healthy adults: a systematic review with meta-analysis. Br J Sports Med.

